# Myotonic Dystrophy: From Molecular Pathogenesis to Therapeutics

**DOI:** 10.3390/ijms231911954

**Published:** 2022-10-08

**Authors:** Lubov Timchenko

**Affiliations:** Departments of Neurology and Pediatrics, Cincinnati Children’s Hospital Medical Center and the University of Cincinnati, Cincinnati, OH 45229, USA; lubov.timchenko@cchmc.org; Tel.: +1-513-803-0768

Current studies concerning myotonic dystrophy type 1 (DM1) are in the process of transitioning from molecular investigations to preclinical and clinical trials. Several papers published in two Special Issues of “Myotonic Dystrophy: From Molecular Pathogenesis to Therapeutics” address different aspects of DM research, focusing on the development of DM1 therapy.

The establishment of DM1 and DM2 therapeutic clinical trials requires more in-depth knowledge regarding the molecular mechanisms behind these diseases, as well as the identification of reliable and quantitative clinical parameters that could be used in the abovementioned clinical trials. This is important, because phenotypes in both diseases are very variable, affecting different tissues and systems with varying severity. Since DM1 is caused by an unstable CTG repeat expansion, the determination of correlations in CTG repeat lengths with clinical outcomes is important. However, multiple symptoms in DM1, as well as their variability from patient to patient, make it difficult to identify specific symptoms, which well correlate with the CTG repeat expansion lengths. A review conducted by Dr. Gourdon’s group [1] discusses the recent knowledge and challenges concerning the genotype/phenotype correlations in DM1. It appears that the association of the CTG repeat lengths with the clinical phenotype could be more informative when DM1 symptoms specific to each DM1 clinical form (congenital, infantile, juvenile, adult and late-onset) are correlated with the length of CTG expansions. Although not perfect, this approach could be useful for the design of clinical trials, selecting patient groups with similar phenotypes. Gourdon’s review also discusses the modifiers that might affect the length of the CTG expansions during intergenerational transmission, including the gender and length of the CTG repeats in the transmitting parent. The paper notes that some discrepancies in studies of the genotype/phenotype correlations in DM1 might occur due to technical difficulties in regard to the size evaluation of the CTG repeat expansions. It seems that the application of small-pool PCR for the evaluation of the inherited length of CTG expansions correlates well with the age of the disease onset in DM1 patients [1,2]. This approach could help better understand the reasons behind the variability of the phenotypes in participants of clinical trials. In addition, Dr. Gourdon’s review addresses the most critical questions asked by researchers and family members with regard to DM1, such as if CTG repeats can contract during parent–child transmission, when CTG repeats would become unstable and what is the role of DNA methylation in the regions of CTG repeats in the increase in the instability of CTG expansions.

While main therapeutic approaches in DM1 focus on the correction of molecular pathophysiology, the best approach would be to prevent the increase in the number of CTG repeats. Multiple factors may affect the increase in CTG instability, including defects in DNA repair and replication, as well as transcription and epigenetic factors. In addition to DNA methylation, mismatched DNA repair proteins have been proposed as modifiers of CTG repeat instability [1,3]. To target the instability of CTG repeats in patients with DM1, the molecular mechanisms of this instability need to be better understood.

Important modifiers of CTG repeat instability are interruptions within CTG expansion (for details, see the review by Dr. Meola’s group [4]). These interruptions might stabilize the CTG expansions and, therefore, they might play a beneficial role in DM1. The presence of CCG, CTC, CAG or other interruptions in CTG expansion in patients with DM1 correlates with the late onset of the disease and overall milder symptoms. This might be due to the increased methylation of the CG-rich regions in the *DMPK* gene. The sequence of interruptions in the CTG expansions, their length and the location in the expansion might have different effects on the disease severity. These factors should also be considered in the evaluation of the genotype/phenotype status in DM1 participants of clinical trials.

Several papers in the Special Issues focus on the development of therapeutic approaches for DM1. One of these approaches is the use of the CRISP/Cas system to cut out the expansion of CTG repeats from the mutant *DMPK* gene, discussed in the review by Dr. Wansink’s group [5] (Figure 1).

Dr. Wansink’s review discusses the current progress with this approach in preclinical studies and potential challenges with the application of the CRISPR-Cas approach in clinical studies. Dr. Wieringa and colleagues also describe the effects of the deletion of CTG repeats using the CRISPR/Cas approach on the improvement of the fusion of DM1 myoblasts, as well as on the global gene expression in the treated DM1 myoblasts [6].

The most rationalized approach for DM1 therapy is the use of antisense oligonucleotides (AONs), degrading the mutant *DMPK* mRNA [13]. Reviews in the Special Issues refer to this approach. While the first attempt to reduce DM1 pathology with the *DMPK1*-specific AON in a clinical trial for adult DM1 was not successful due to the low penetration of AON in skeletal muscle, studies on the improvement of this approach continue. More importantly, preclinical studies in DM1 showed that AONs were beneficial not only for the skeletal muscle pathology, but that they could also reduce cognitive defects and reverse cardiac phenotypes [12,14,15]. As described in Dr. Mahadevan’s review, which focused on cardiac pathology in DM [12], the application of the *DMPK*-specific AON in the preclinical study using a tet-inducible DM1 mouse model, expressing the 3′UTR of *DMPK* with 200 CUG repeats, reduced skeletal and cardiac muscle deficiencies [12,15]. This paper provides a comprehensive discussion in regard to the cardiac phenotype in DM1 patients, the possible molecular pathogenesis in DM1 hearts and in vivo models to study DM1-linked cardiac deficiency. The authors also discuss the recommended clinical guidelines for the management of cardiac pathology in DM1 and the application of cardiac magnetic resonance imaging as a biomarker for cardiac deficiency in patients with DM1. This knowledge is important for DM1clinical trials.

Numerous reports showed that the expanded CUG repeats affect several RNA CUG-binding proteins, including MBNL1 and CUGBP1. MBNL1 is sequestered by CUG repeats and, as a result, MBNL1 activity is reduced in DM1. The review by Dr. Swanson’s group describes the role of MBNL proteins in the disruption of RNA processing in DM1 and DM2 [16]. The authors discussed several possible mechanisms of the MBNL1-CUG or CCUG foci assembly in DM1 and DM2 cells, and the effect of MBNL1 sequestration on RNA processing.

CUGBP1 was identified as the first CUG RNA-binding protein, playing an essential role in DM1 pathology [9]. One of the papers in the Special Issue discusses the contribution of CUGBP1 in DM1 pathogenesis [9]. While this protein is increased in DM1 (similar to several other RNA-binding proteins), its activity in DM1 patients is altered on several levels. Thus, the recovery of the activities of CUGBP1 in DM1 should include the correction of CUGBP1 levels and the normalization of CUGBP1 activity. Since CUGBP1 activity is regulated by GSK3β through the GSK3β-cyclin D3-CDK4 pathway, the inhibitors of GSK3 could be used to restore CUGBP1 activity. As discussed, a small inhibitor of GSK3, tideglusib, reduced skeletal muscle and cognitive defects in DM1 mouse models (*HSA^LR^* and DMSXL mice) [9]. This small molecule also reduced neuromuscular and cognitive abnormalities in patients with CDM1 in a phase II clinical trial [17].

Two reviews by Dr. Berglund’s and Dr. Brook’s groups focused on identified or synthesized small molecules correcting MBNL1 and CUGBP1 [7,10]. Among the small molecules, which could be used as potential therapeutics, are kinase inhibitors [10]. The Special Issue also shows that microRNAs could be used to normalize MBNL1 and CUGBP1 in DM1 [11]. MicroRNAs altered in DM1 could also be used as biomarkers. The review by Dr. Munain’s group describes the recent data related to the potential role of small-molecule metformin for the treatment of DM1 [8]. Some studies showed that metformin improves the splicing of insulin receptors, misregulated in DM1. Therefore, the role of metformin was tested in regard to the correction of insulin resistance in DM1. However, it turns out that metformin may affect several splicing events in DM1, reducing myotonia. Thus, the benefits of this antidiabetic drug are currently being tested in the clinical trial for patients with adult DM1. Recent data suggested that metformin could improve the mobility of patients with DM1 (reviewed in [8]).

Drug testing in DM1 and DM2 requires appropriate models, such as fly and mouse, with DMSXL mice [18] being one of the models commonly used in DM1 preclinical studies. The advantage of this model is that these mice express long CUG repeats within the human *DMPK* gene under the *DMPK* promoter. As a result, the mutant CUG repeats can affect not only skeletal muscle such as in *HSA^LR^* mice [19], but also the heart and CNS function. Dr. Gomes-Pereiro’s group characterized the CNS phenotype in DMSXL mice and found abnormal glutamate and GABA neurotransmission and altered synaptic plasticity [18]. Thus, this mouse model could be used for the testing of therapeutics improving cognitive abnormalities in DM1.

The review by Dr. Artero’s group summarizes multiple “omics” data, produced in DM studies, including a microarray analysis, RNA-seq, high-throughput sequencing of cross-linked immunoprecipitates (HITS-CLIP) of MBNL proteins and CUGBP1 and other tests [20]. These data are critical for the understanding of molecular pathogeneses in DM1 and DM2, as well as for the analysis of the molecular mechanisms of potential drugs in preclinical and clinical studies.

Thus, the papers in the Special Issues on DM1 reveal important progress in the development of the potential therapeutic approaches in DM1, and discuss molecular and in vivo findings required for future successful clinical studies.

## Figures and Tables

**Figure 1:**
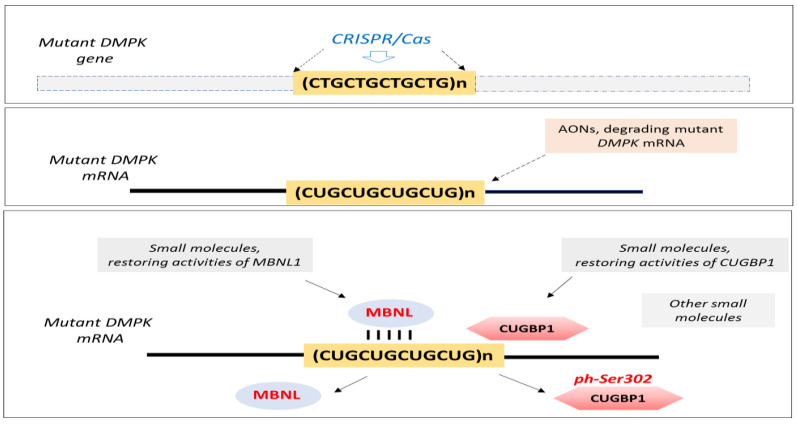
Therapeutic approaches for DM1 discussed in the reviews in the Special Issues of the journal. They include the CRISPR/Cas approach [5,6], as well as the application of small molecules, restoring MBNL1 and CUGBP1 activities or correcting splicing via other RNA-binding proteins [7,8,9,10]. Although indirectly, several review papers referred to the use of AONs as a potential therapy for DM1 [5,7,8,10,11,12].
